# A Disease Marker for Aspirin-Induced Chronic Urticaria

**DOI:** 10.3390/ijms150712591

**Published:** 2014-07-15

**Authors:** Chia-Wei Hsieh, Jeen-Wei Lee, En-Chih Liao, Jaw-Ji Tsai

**Affiliations:** 1 Division of Allergy, Immunology and Rheumatology, Department of Internal Medicine, Taichung Veterans General Hospital, No. 1650, Sec. 4, Taiwan Boulevard, Taichung 40705, Taiwan; E-Mail: chiaweih@gmail.com; 2 Division of Allergy, Immunology and Rheumatology, Department of Internal Medicine, Wei Gong Memorial Hospital, No. 128, Xinyi Rd., Toufen Township, Miaoli County 35159, Taiwan; E-Mail: leelawrence1688@gmail.com; 3 Center for Translational Medicine, Department of Medical Research, Taichung Veterans General Hospital, No. 1650, Sec. 4, Taiwan Boulevard, Taichung 40705, Taiwan; E-Mail: en65@vghtc.gov.tw; 4 Department of Medicine, Chung-Shan Medical University, No. 110, Sec. 1, Jianguo N. Rd., Taichung 40201, Taiwan; 5 Faculty of Medicine, National Yang Ming University, No. 155, Sec. 2, Linong Street, Taipei 11221, Taiwan

**Keywords:** aspirin hypersensitivity, aspirin induced chronic urticaria, CD203c, high-affinitiy IgE receptor, single nucleotide polymorphism, basophil activation activity

## Abstract

There are currently no diagnostic methods *in vitro* for aspirin-induced chronic urticaria (AICU) except for the provocation test *in vivo*. To identify disease markers for AICU, we investigated the single nucleotide polymorphism (SNP) of the promoter loci of high-affinity IgE receptor (*FcεRIα*) and CD203c expression level in Chinese patients with AICU. We studied two genotypic and allelic frequencies of rs2427827 (–344C/T) and rs2251746 (–66T/C) gene polymorphisms of *FcεRIα* in 20 patients with AICU, 52 subjects with airway hypersensitivity without aspirin intolerance, and 50 controls in a Chinese population. The results showed that the frequencies of two SNPs (–344C>T, –66C>T) were similar to the normal controls. The allele frequency of –344CC was significantly higher in the patients with AICU compared to those with airway sensitivity (*p* = 0.019). We also studied both histamine release and CD203c expression on KU812 cells to assess aspirin-induced basophil activation. We found that the activity of basophil activation of AICU was significantly higher in the patients with AICU compared to those with airway hypersensitivity without aspirin intolerance. The mean fluorescence intensity of the CD203c expression were 122.5 ± 5.2 *vs.* 103.3 ± 3.3 respectively, (*p* < 0.05), and the percentages of histamine release were 31.3% ± 7.4% *vs.* −24.0% ± 17.5%, (*p* < 0.05) respectively. Although the mean fluorescence intensity of CD203c expression and the percentage of histamine release were significantly up-regulated by aspirin, they were not affected by anti-IgE antibodies. These results suggest that a single SNP of *FcεRIα* (–344C>T) is less likely to develop AICU and the basophil activation activity in the sera by measuring CD203c expression can be applicable to confirm the diagnosis of AICU.

## 1. Introduction

Chronic urticaria (CU) is a common allergic disease [1]. Although its pathogenic mechanisms remain unclear, mast cell activation and the release of histamine are thought to play a central role [2,3]. Patients with atopy have a greater likelihood of having aspirin hypersensitivity, including aspirin-induced asthma and/or aspirin-induced chronic urticaria [3,4,5]. We hypothesized that the early detection of aspirin hypersensitivity among atopic patients should be able to reduce the risk of CU. Up to 6%–30% of patients with CU experience flares of hives following the ingestion of aspirin or chemically unrelated non-steroidal anti-inflammatory drugs (NSAIDs) [6,7,8,9,10]. However, to the best of our knowledge, there are currently no established methods *in vitro* to diagnose NSAID-induced CU except for provocation with NSAIDs *in vivo*. Aspirin is one of the most commonly used NSAIDs, and it is therefore important to identify disease markers and prevent the development of aspirin-induced CU (AICU).

The pathogenesis of AICU is thought to involve two different pathways, including IgE-mediated and non-IgE mediated pathways. Urticaria, angioedema and respiratory distress are the main clinical presentations of immediate-type aspirin-induced hypersensitivity. IgE-mediated activation of basophils and mast cells has been proposed to play an important role in the pathophysiology of immediate-type drug hypersensitivity [11]. Upon binding of allergens to IgE on the IgE receptor, and especially the high-affinity IgE receptor (FcεRIα), basophils/mast cells immediately release immune mediators including histamine, leukotrienes, prostaglandin D2, cytokines, and proteolytic enzymes [12,13]. The IgE receptor is a tetramer composed of a ligand-binding α-chain, a signal augmenting β-chain, and a signal transducing γ-chain [11]. The mechanism of AICU is not completely understood, and specific IgE to aspirin is usually not detectable [1]. As in the position paper of the European academy of allergy and Clinical Immunology, AICU can be included in the classification of NSAIDs-exacerbated cutaneous disease (NECD) and it was thought to be associated to COX-1 inhibition [14]. Some authors found that the genetic polymorphisms on the α-chain of the high-affinity IgE receptor may contribute to the development of AICU and also have the role in IgE-mediated histamine release [3]. In addition, there also have reported that the clinical efficacy of anti-IgE antibody in idiopathic chronic urticaria [15]. Therefore, it is feasible to investigate the role of IgE in the pathogenesis of AICU. Furthermore, there are currently no established and reliable techniques to diagnose AICU. Apart from medical history, the most reliable method of diagnosis is re-challenge with aspirin, which may induce severe clinical reactions.

The basophil activation test has proven to be a useful tool for the assessment of immediate-type responses to allergens mediated by IgE. In addition, different activation markers (CD203c or CD63) with distinct modes of regulation have been used to evaluate the basophil response to allergens. Although flow cytometric detection of basophil activation by CD63 expression has been studied in diagnosing immediate-type allergies to various allergens such as aeroallergens (cypress pollen, house dust mites), foods, hymenoptera venom, natural rubber latex and drugs [16], CD203c expression is still considered to be the most useful marker of basophil activation. The basophil activation test has recently been updated [17], and the CD203c expression on human basophils has been reported to be associated with asthma exacerbation [18]. Basophil histamine release has also been reported to be correlated with the symptoms of hypersensitivity reactions, and that it may be used to replace the allergen skin test which is less specific to the allergens [19]. CU was reported to be associated with basophil activation and CD203c expression and histamine release can be a diagnostic method *in vitro*. Since the symptoms of acute exacerbation are similar to IgE- medicated reaction and aspirin-induced acute angioedema has been case reported due to IgE [16]. It is feasible to compare the basophil reaction using aspirin and serum in the presence of anti-IgE antibodies. FcεRIα promoter polymorphisms have been found to be associated with AICU, and a single nucleotide polymorphism (SNP) of FcεRIα has been reported in aspirin-intolerant asthma and is considered to be an important regulator of IgE serum level in allergic subjects [20,21,22]. However, the role of FcεRIα promoter polymorphisms in basophil activation leading to an increase in histamine release and CD203c expression has not been addressed in these studies.

The aim of this study was to identify specific disease markers to diagnose AICU using cells and serum from patients with AICU and analyzing the promoter genes of FcεRIα and measuring basophil activation activity *in vitro*. In addition, we hypothesized that the effect of anti-IgE antibodies on IgE-mediated basophil activation may help to clarify the role of IgE in the pathogenesis of AICU.

## 2. Results

The general data, serum total IgE and mite-specific IgE of the patients with AICU and control groups using for analysis of CD203c and histamine release are summarized in Table 1.

**Table 1:** Disease characteristics of the subjects with aspirin-induced chronic urticaria (AICU) and control group with those not allergic to aspirin.

Parameter	AICU (*n* = 19)	Control Group (*n* = 12)	*p* Value
Mean age * (years)	40.63 ± 3.52	36.67 ± 5.66	0.53
Female gender (%)	10 (52.63%)	10 (83.33%)	<0.01
Total IgE ^†^ (IU/mL)	110	96.85	^#^ NS
% of Mite Cap positive	73.68 (14/19)	41.67 (5/12)	0.08
Airway allergy (*n*, present/total)	8 (42.1%)	3 (25%)	0.35

*, Mean ± SEM; ^†^, Median; ^#^ NS, not significant.

### 2.1. The Genotype and Allele Frequency of the α-Chain of High-Affinity IgE Receptor (FcεR1α) in Aspirin-Induced Chronic Urticaria (AICU)

Two polymorphisms of FcεR1α (–344C>T, –66C>T) were genotyped in 20 AICU patients and compared to 50 normal healthy controls and 52 subjects with airway hypersensitivity without aspirin intolerance. The frequencies of both polymorphisms were similar to the normal controls, however the allele frequency of –344CC was significantly higher in the patients with AICU compared to those with airway hypersensitivity without aspirin intolerance (*p* = 0.019) (Table 2). This result excludes the role of FcεRIα polymorphism (–344C>T) in AICU patients, and may be more specific for IgE-related airway allergies.

**Table 2:** The genotype and allele frequency of the promoter gene of *FcεR1α* (FcεR1α –66T>C, rs2251746 and –334C>T, rs242782).

Loci and Genotype	AICU (*n* = 20)	Normal Controls (NC) (*n* = 50)	Airway Allergies (AA) (*n* = 52)	* p Value	
				AICU *vs.* NC	AICU *vs.* AA
FcεR1α –66T>C					
TT	19 (95.0%)	38 (76.0%)	42 (80.8%)		
TC	1 (5.0%)	12 (24.0%)	10 (19.2%)	0.091	0.27
^†^ H-W-P	<0.001	0.864	0.724		
FcεR1α –344C>T					
CC	20 (100%)	40 (80%)	36 (69.2%)	0.097	0.019
CT	0 (0.0%)	9 (18%)	9 (17.3%)		
TT	0 (0.0%)	1 (2%)	7 (13.5%)		
^†^ H-W-P	<0.001	0.850	0.002		

* by the chi-square test; ^†^ H-W-P, *p* value for the Hardy–Weinberg equilibrium patients in question.

### 2.2. Differences in Basophil Activation Activity in the Sera between the Patients with AICU and Control Group

Differences in basophil activation activity in the sera between the patients with AICU (*n* = 17) and control group (*n* = 11) were compared by CD203c expression and histamine release. KU812 cells were incubated with aspirin and the patient’s sera in the presence or absence of anti-IgE antibody followed by the measurement of CD203c expression and histamine release. Although the MFI of CD203 expression was similar between the two groups, the expression was only significantly up-regulated by aspirin in the subjects with AICU (108.6 ± 2.8 *vs.* 122.5 ± 5.2) and not in control group (107.0 ± 5.1 *vs.* 103.3 ± 3.1). The up-regulated CD203c expression by aspirin was not affected by anti-IgE antibody (Table 3). These results indicated that the up-regulation of CD203c and histamine release by aspirin was not IgE mediated. The percentage of histamine release was also higher in the subjects with AICU compared with control group (31.3% ± 7.4% *vs.* −24.0% ± 17.5%; *p* < 0.05) (Table 3). For evaluating KU812 cells using in expression of CD203c and basophil histamine releasing assay, we performed ROC curve analysis (AUC: 0.813, *p* < 0.001) and the sensitivity and specificity were 75% and 80%, respectively. Although the histamine release was down-regulated by anti-IgE, it did not reach a statistically significant level.

**Table 3:** Differences in basophil activation activity in sera between the subjects with AICU and those not allergic to aspirin.

Factors	AICU (*n* = 17)			Not Allergic to Aspirin (*n* = 11)		
Aspirin	−	+	+	−	+	+
Anti-IgE antibody	−	−	+	−	−	+
Histamine release (%) ^†^		31.3 ± 7.4 ^a^	1.6 ± 12.3		−24.0 ± 17.5 ^b^	−57.3 ± 24.9
CD203c expression (MFI)	108.6 ± 2.8 ^c^	122.5 ± 5.2 ^d^	120.2 ± 6.7	107.0 ± 5.1 ^e^	103.3 ± 3.3 ^f^	99.2 ± 3.1

^†^, 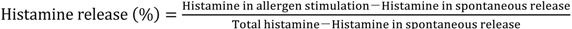; Data presented as mean ± SEM; ^a,b^, *p* < 0.05 analyzed by the Mann–Whitney U test; ^c,d^, *p* < 0.05 analyzed by the Mann–Whitney U test; ^d,f^, *p* < 0.05 in comparison with control by the *t*-test; ^e,f^, not significant.

### 2.3. Differences in Basophil Activation Activity in the Sera between Subjects with AICU with and without Airway Allergy

Differences in basophil activation activity in the sera of patients with AICU (*n* = 7) with and without airway allergy were compared. Basophils were incubated with aspirin and the patient’s sera in the presence or absence of anti-IgE antibody, followed by measurement of CD203c expression and histamine release. The MFI of CD203c expression was up-regulated by aspirin in both groups, however, there was no significant up-regulation in those without airway allergy. The up-regulation of MFI in both groups was not affected by anti-IgE antibody (Table 4). The significant up-regulation of CD203c was also not inhibited by anti-IgE antibody, suggesting that basophil activation activity in the sera was not mediated by IgE. There was also no statistical significance in histamine release between the subjects with and without airway allergy, despite the up-regulation of CD203c expression in those with airway allergy (Table 4). Although the histamine release was decreased by anti-IgE antibody, there was no statistically significant inhibition by anti-IgE in either group.

**Table 4:** Differences in basophil activation activity in sera between subjects with AICU with and without airway allergy.

Factors	Airway Allergy (*n* = 7)			No Airway Allergy (*n* = 10)		
Aspirin	−	+	+	−	+	+
Anti-IgE antibody	−	−	+	−	−	+
Histamine release (%) ^†^	−	30.8 ± 8.1 ^a^	9.3 ± 4.4 ^g^	−	31.7 ± 11.6 ^b^	−3.7 ± 21.1 ^h^
CD203c expression (MFI)	102.7 ± 3.2 ^c^	115.9 ± 6.4 ^d^	115.2 ± 7.0	113.2 ± 3.7 ^e^	127.6 ± 7.6 ^f^	124.1 ± 10.8

^†^, 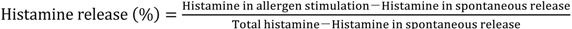; Data presented as mean ± SEM; ^a,b^, not significant; ^c,d^, *p* < 0.05 in comparison with medium control by the *t*-test; ^e,f^, not significant; ^g,h^, *p* < 0.05 in comparison with medium control by *t*-test. ^a,g^, not significant; ^b,h^, not significant.

### 2.4. Differences in Basophil Activation Activity between the Subjects with AICU with and without Elevation of Total IgE or Mite Specific-IgE

**Figure 1:**
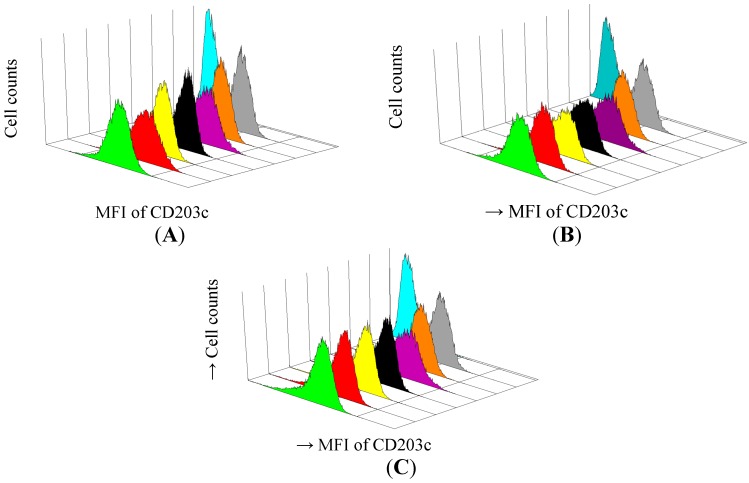
Basophil activation assay measuring activation marker CD203c on basophils of patients AICU with IgE-mediated response (**A**) AICU without IgE-mediated response (**B**) and normal healthy controls (**C**). Flow cytometric quantification of de-granulated basophils on cell surface marker CD203c. Data presented as mean fluorescence intensity of CD203c of three cases.

Differences in basophil activation activity between the subjects with AICU with or without elevation of total IgE were compared. Anti-IgE was used to determine whether it could block the effect of serum-IgE mediated basophil activation activity in the serum. Basophils were incubated with aspirin and the patient’s sera in the presence or absence of anti-IgE antibody followed by the measurement of CD203c expression and histamine release (Figure 1). The results showed that CD203c expression was down-regulated by anti-IgE in some patients as analyzed by histograms, indicating it was an IgE-mediated response (Figure 1A), CD203c expression was not down-regulated by anti-IgE antibody treatment in most cases (Figure 1B), indicating it was not an IgE-mediated response. Although the MFI of CD203c was up-regulated significantly by aspirin in both groups of patients, there were no statistically significant differences between the groups. The up-regulation of MFI in both groups was not affected by anti-IgE antibody (Table 5A). Similar findings were observed in the subjects with and without elevation of mite-specific-IgE, and there were no statistically significant difference between both groups. When histamine release was compared, there were no statistically significant difference between the subjects with and without elevation of total IgE (Table 5A) and mite-specific-IgE (Table 5B). Although histamine release was decreased by anti-IgE antibodies, there was no statistically significant inhibition by anti-IgE antibodies.

: serum + anti-IgE antibody + aspirin; : serum + aspirin; : serum; ■ 4: serum + anti-IgE antibody; : A23187 (positive control); : anti-IgE antibody; : aspirin; : KU812 cell.

**Table 5:** Differences in basophil activation activity between the patients with AICU with or without elevation of total (**A**) or mite-specific (**B**) IgE.

**(A) Total IgE**	**≥400 KU/mL (n = 6)**			**<400 KU/mL (n = 11)**		
Aspirin	−	+	+	−	+	+
Anti-IgE antibody	−	−	+	−	−	+
Histamine release (%) ^†^		31.7 ± 8.6 ^a^	8.6 ± 7.5		34.6 ± 9.6 ^b^	2.2 ± 18.9
CD203c expression (MFI)	106.5 ± 2.0 ^c^	120.4 ± 11.2 ^d^	121.0 ±11.0	109.3 ± 3.7 ^e^	123.2 ± 6.1 ^f^	119.9 ± 18.4
**(B) Mite-specific IgE**	**≥0.35 KU/mL (*n* = 13)**			**<0.35 KU/mL (*n* = 4)**		
Histamine release (%) ^†^		30.5 ± 8.8 ^a^	1.8 ± 12.8		34.0 ± 15.4 ^b^	1.1 ± 36.3
CD203c expression (MFI)	108.0 ± 3.5 ^c^	120.9 ± 5.6 ^d^	123.3 ± 8.8	110.4 ± 4.5 ^e^	127.4 ± 13.4 ^f^	114.4 ± 6.6

^†^, 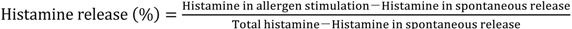; Data presented as mean ± SEM; ^a,b^, not significant; ^c,d^, *p* < 0.05 in comparison with medium control by the *t*-test; ^e,f^, *p* < 0.05 in comparison with medium control by the *t*-test.

## 3. Discussion

A few genetic markers have been suggested for the detection of aspirin-exacerbated respiratory diseases. However, genetic studies on AICU are limited. Some reports have indicated possible associations between genetic polymorphisms and responses to aspirin hypersensitivity [23]. Shikanai *et al.* described three promoter polymorphisms in the *FcεRIα* gene, namely –770A>C, –664G>A, and –335C>T in Caucasian and African Americans with asthma [24]. The polymorphism –66T>C in the *FcεRIα* gene has been reported in Japanese patients with atopic dermatitis [20]. Bae *et al.* reported two SNPs at –344C>T and –95T>C in Korean patients with AICU [3]. In this study, we analyzed these two polymorphisms in our patients with AICU, and the results showed that both genotype allele frequencies were similar to those of the normal controls. Although the allele frequency of –344C>T was higher than in those with allergic disease, further investigations with more patients are needed to confirm whether –344C>T can be used to exclude AICU in subjects with airway allergy. Similarly, Zhang *et al.* reported that –95T>C was not associated with allergic rhinitis [21]. This suggests that genetic variants in *FcεRIα* which may serve as a useful marker to predict AICU may vary by ethnicity. The importance of ethnicity in genetic studies has been recently highlighted by Kim *et al.* [25].

The IgE-dependent activation of human basophils on histamine release and up-regulation of CD203c has been widely used for the clinical diagnosis of systemic mastocytosis in patients with medication-related symptoms. In our study, the up-regulation of CD203c expression and histamine release were detected in all of the AICU patients taking aspirin. Anti-IgE antibody treatment did not interfere with the expression of CD203c on basophils, however it did decrease histamine release. This result indicates that the serum factors involved in aspirin-induced histamine expression are not mediated by IgE. The discrepancy between histamine release and CD203c expression is not uncommon, since histamine releasing factors in sera have been reported [26].

Our results showed that there was no difference in the activity of basophil activation between the patients with AICU with or without elevated serum total IgE. Although, anti-IgE antibodies were found to counteract with serum IgE in some cases, the results indicate that IgE only plays a partial role in the basophil activation causing mediator release in AICU. Since the histamine release activity in the sera was not related to IgE, other factors in the sera of our AICU patients may also have played a role. Since histamine release factors in sera have been reported in the sera of patients with CU, this issue cannot be clarified until the antibody to histamine release factor has been identified.

Using basophil cell line KU812 cells for both CD203c expression and histamine release could be used to determine the basophil activation activity in the sera of patients with AICU, and to identify the AICU patients using *in vitro* tests. This is the first study to identify AICU using sera and KU812 cells *in vitro* in the presence or absence of aspirin via the analysis of CD203c expression and histamine release activity, and this is a more sensitive method compared to using purified peripheral basophils to identify AICU.

The major limitations of this study are that only 20 AICU patients were included in the study, and no purified peripheral basophils derived from the patients were used. Since there are only a few percent of basophils were present in the peripheral blood, and fresh purified basophils are more easily activated during preparation. However, basophil cell line KU812 cells helped to offset this limitation, as using the cell line avoided pre-bound IgE on basophils and also provided an adequate amount of basophils for the study. In addition, using a cell line avoids the inconvenience of cell preparation and prevents unstable basophil isolation from AICU patients especially those patients still receive treatment and under medication, and is therefore a good alternative choice. As limited number of subjects in our preliminary study, further larger scale investigation should be performed for further confirmation.

## 4. Material and Methods

### 4.1. Study Subjects

The Institutional Review Board of Taichung Veterans General Hospital reviewed and approved the ethical issues of this study (file number C05213). Written informed consent was obtained from each participant before they were enrolled into this study. A total of 20 AICU subjects and 52 subjects with airway hypersensitivity without aspirin intolerance who attended the Allergy Clinic at Taichung Veterans General Hospital were recruited for this study. AICU was defined as a history of chronic urticaria for more than 6 weeks and no other known causes of chronic urticaria. The exacerbation of urticaria was proved by aspirin provocation test with 500 mg aspirin. Airway hypersensitivity was defined as having a history of recurrent nasal stuffiness, nasal itching, sneezing, rhinorrhea, and/or asthma as defined by Allergic Rhinitis and its Impact on Asthma (ARIA) guidelines [27]. Normal controls were recruited from the general population. Control group was defined as no personal or family history of allergic diseases, including food allergy, allergic rhinitis, atopic dermatitis, and no aspirin or other drug hypersensitivity. A total of 20 patients with AICU, 52 patients with airway hypersensitivity and 50 subjects as normal control were enrolled in genetic association study. Peripheral blood samples (10 mL) were collected with EDTA syringe and DNA from the buffy coat was purified using a Genomic DNA Mini kit (Geneaid, Taoyuan, Taiwan). Sera were stored at −70 °C before assessment.

### 4.2. Serum Total IgE and Mite-Specific IgE

Because mite allergens commonly affect allergic patients, total IgE and specific IgE to mite concentrations were measured using a UniCAP system (Thermo Fisher Scientific, Uppsala, Sweden) as a background value.

### 4.3. Genotyping by Sequencing

A set of primers was designed for the amplification and sequencing based on GenBank sequences. The forward and reverse primer sequences for the FcɛRIα promoter region were 5'-AAgAAAAgCgTTggTAgCTCTggTg-3' and 5'-ATCTTCTTCATggACTCCTggTgC-3', respectively. Sequence variants were verified using chromatograms on an ABI 3730 DNA analyzer (Applied Biosystems, Foster City, CA, USA). Two SNPs in the promoter region were identified at –344 (rs2427827) and –66 (rs2251746).

### 4.4. Basophil Histamine Release Assay

The sera from 19 patients with AICU and 12 aspirin-tolerant control subjects were collected for basophil histamine release assay. The patients were instructed to avoid the use of systemic antiallergenic drugs (including antihistamine and systemic corticosteroid drugs) for at least 24 h before blood sampling. We excluded those subjects with pre-medications, there were only 17 out of 19 patient with AICU and 11 out of 12 subjects in control group were performed histamine releasing assay.

KU812 cells (human basophilic leukemia cell line, purchased from Riken Cell Bank, Tsukuba, Japan) were resuspended in medium RPMI-1640 by adjusting to 1 × 10^6^ cells/mL using trypan blue. Passive sensitization of the KU812 cells with sera from the patients or controls (1/5 the volume of subjects’ sera) was performed for 4 h at 37 °C. Anti-IgE antibody (Xolair, Novartis Pharma AG, Stein, Switzerland) was added to detect the role of IgE-mediated basophile activation in the patients with AICU. The anti-IgE antibody selectively binds to free human IgE in the blood and to the membrane-bound form of IgE (mIgE) on the surface of mIgE-expressing B lymphocytes. Before the passive sensitization, the anti-IgE antibody was added and mixed with the sera at a concentration of 100 μg/mL for 30 min. After incubation with the sera, the sensitized cells were then incubated with 100 μg/mL of aspirin (Stin, China Chemical, Taiwan) for 30 min at 37 °C, and with 1 μg/mL of A23187 (Sigma-Aldrich, St Louis, MO, USA) for total release histamine, and then the supernatant was collected and reacted with *O*-phthalaldehyde (OPA, 5 mM) for 7 min. The reaction was stopped by adding H_2_SO_4_ (0.04 M). The histamine released into the supernatant was measured by a fluorescence spectrophotometer, and the percentage of histamine release was calculated by the following formula: (stimulated released histamine—Spontaneously released histamine)/(total released histamine—Spontaneously released histamine) × 100%.

### 4.5. CD203c Expression on Basophils

The sera from the same donors for the basophil histamine release assay were collected. KU812 cells were resuspended in medium RPMI-1640 by adjusting to 1 × 10^6^ cells/mL using trypan blue. Passive sensitization of KU812 cells with sera from the patients or controls (1/10 the volume of subjects’ sera) was performed for 4 h at 37 °C. Anti-IgE antibody (Omalizumab, Xolair, Novartis Pharma AG, Stein, Switzerland) was added to detect the role of IgE-mediated basophile activation in the patients with AICU. Before the passive sensitization, the anti-IgE antibody was added and mixed with the sera at a concentration of 100 μg/mL for 30 min. After incubation with the sera, the sensitized cells were then incubated with 100 μg/mL of aspirin (Acetylsalicylic acid, Stin, China Chemical, Taipei, Taiwan) for 30 min at 37 °C, and with 1 μg/mL of A23187 (Sigma) for maximum CD203c expression. The cells was collected and suspended in PBS, and then reacted with FITC-conjugated anti-CD203c mAb (Pharmigen, Germany) for 30 min at 4 °C. All of the samples were centrifuged (300 g, 5 min at 4 °C) and analyzed within 2 h on a FACScan flow cytometer (Becton Dickinson Diagnostic System, San Jose, CA, USA). At least 10,000 basophilsper tube were acquired, and the expression of CD203c was analyzed on the gated cell population. The results were given as mean fluorescence intensity (MFI) of basophil expression.

### 4.6. Statistical Analysis

Statistical analysis was performed using SPSS software (SPSS version 16.0, SPSS Inc., Chicago, IL, USA). The genotype frequencies of each SNP were examined for significant departures from Hardy-Weinberg equilibrium using χ^2^ tests. Differences in genotype frequency between the patients and controls were also analyzed using χ^2^ tests. Differences in histamine release between the patients with AICU and those who were not allergic to aspirin were examined using the Mann–Whitney U test. A *p* value of 0.05 or less was regarded as indicating significance. Statistical comparisons of the CD203c expression between the patients and controls were performed using paired *t*-tests. Sensitivity, specificity and area under the curves (AUC) were calculated for KU812 cells using in expression of CD203c and basophil histamine releasing assay.

## 5. Conclusions

Patients may not be aware of aspirin intolerance and the association between the use of NSAIDs and AICU exacerbations. Although provocation *in vivo* using various forms of oral aspirin have been used to diagnose AICU for many years, it is not practical due to unpredictable severe systemic reactions. This study demonstrates that patients with a single SNP of *FcεRIα* (–344C>T) are less likely to develop AICU, and that CD203c expression by basophil activation activity in sera can confirm the diagnosis of AICU. These *in vitro* disease markers may be a useful tool for assessing aspirin hypersensitivity in patients with chronic urticaria.
